# Molecular dynamics of rolling and twisting motion of amorphous nanoparticles

**DOI:** 10.1038/s41598-021-93984-1

**Published:** 2021-07-16

**Authors:** Philipp Umstätter, Herbert M. Urbassek

**Affiliations:** Physics Department and Research Center OPTIMAS, University Kaiserslautern, Erwin-Schrödinger-Straße, 67663 Kaiserslautern, Germany

**Keywords:** Atomistic models, Astrophysical dust

## Abstract

Granular mechanics codes use macroscopic laws to describe the damping of rolling and twisting motion in granular ensembles. We employ molecular dynamics simulation of amorphous Lennard–Jones grains to explore the applicability of these laws for nm-sized particles. We find the adhesive force to be linear in the intergrain attraction, as in the macroscopic theory. However, the damping torque of rolling motion is strongly superlinear in the intergrain attraction. This is caused by the strong increase of the ‘lever arm’ responsible for the damping torque—characterizing the asymmetry of the adhesive neck during rolling motion—with the surface energy of the grains. Also the damping torque of twisting motion follows the macroscopic theory based on sliding friction, which predicts the torque to increase whit the cube of the contact radius; here the dynamic increase of the contact radius with angular velocity is taken into account.

## Introduction

The atomistic nature of nanosized particle contacts is of relevance in several areas of science and technology, including tribology and cluster deposition technology^1^. In these areas, atomistic simulations have already been intensely used to understand contact processes, such as sliding friction^2–6^ or perpendicular forces during contacts^7,8^.

**Figure 1:**
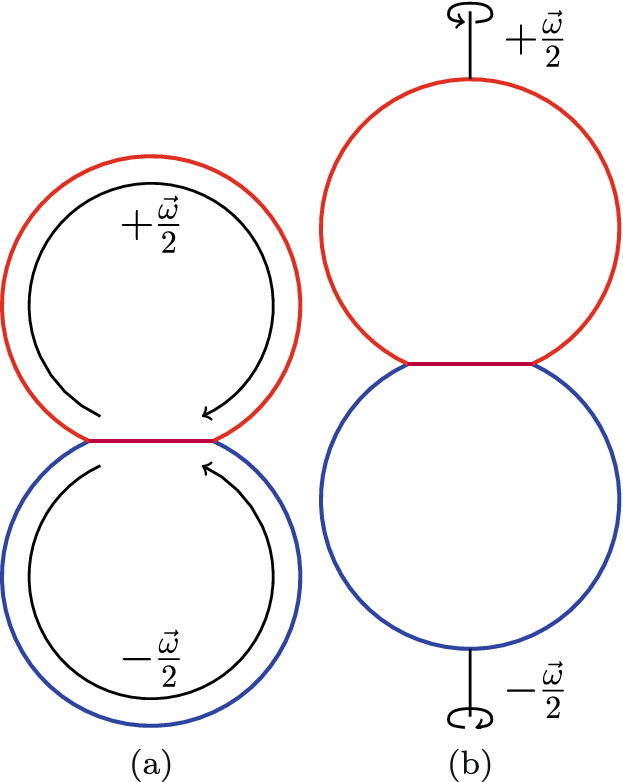
Schematic of (**a**) rolling and (**b**) twisting rotation of two grains in contact.

Particle contacts also play a fundamental role in all aspects of granular mechanics^9^. This field uses modeling and simulation to describe the dynamics of granular materials and requires rules to model the interaction of grains. Even if grains are assumed to be spherical, the interactions between two grains are complex. Besides the normal force exerted during contact, tangential forces and torques act during the collision. The understanding of the normal forces is often based on viscoelastic extensions of Hertz’ contact law, see for example^10^; an attractive part based on Johnson–Kendall–Roberts (JKR)^11^ or Derjaguin–Muller–Toporov (DMT) theory^12^ is added. Tangential forces arise from sliding friction of the contacting grains in the contact plane. Rotational motion of the grains is changed during contact by torques exerted. Depending on the component of the grain’s angular momentum parallel or perpendicular to the contact plane, one may talk about rolling or twisting grain rotation, respectively, see Fig. 1. Dominik and Tielens^13^ provide an overview over these elementary mechanical contact forces and torques, as they are used in several granular mechanics models and collision codes^10,14–17^ and for the analysis of collision experiments between spherical grains^18–22^.

These models are primarily obtained from macroscopic contact mechanics based on continuum equations. However, in the description of the contact, invariably atomistic concepts enter, for instance in the consideration of the role of surface steps on the generation of rolling torques^23,24^, in the models underlying sliding friction and twisting torques^25^, or in the modeling of the adhesive neck forming in the contact zone. It is of interest to study the atomistic basis underlying these contact models. In addition, for collisions of nanoparticles, the derivation of the laws from a continuum description of the contact becomes dubious, as interatomic distances are not negligible compared to the size of contacts in this regime, and therefore requests an atomistic analysis^26^.

Parameters derived from the collisional models are used in the description of complex granular materials, such as the collision of granular aggregates. Thus, the bouncing (or sticking) velocity describes the minimum relative velocity of two grains needed to bounce from each other after the collision^13,27^ and hence describes aggregate destruction. The rolling energy is the minimum energy a grain needs to roll an angle of $90^\circ$ over another grain; this energy is believed to describe collisional restructuring processes^13,27^. As dissipative processes—damping torques in the case of rotation—enter these characteristic energies, a critical evaluation of the atomistic nature of these processes is also important for these collision parameters.

For rolling and twisting motion of nanoparticles, there seem to be no studies available that compare the predictions of macroscopic contact models of granular mechanics with the results of atomistic simulations. The present paper aims to fill this gap using dedicated molecular dynamics simulations. To this end, we study the torques generated in the contact zone of two grains that are rotating in their contact. Since we are interested in the generic behavior of these grains, rather than in specific materials, we use a Lennard–Jones potential for their description. These grains are nm-sized so that we can analyze the damping of their motion using molecular dynamics simulation. By comparing to macroscopic laws, we can evaluate their validity in describing nanoparticle rotational motion.

## Method

In our molecular dynamics simulations we use spherical grains containing 100,000 atoms. Atoms interact via the Lennard–Jones (LJ) potential,

1 $$\begin{aligned} V(r) = 4 \epsilon \left[ \left( \frac{\sigma }{r} \right) ^{12} - \left( \frac{\sigma }{r} \right) ^{6} \right] , \end{aligned}$$

cut off at $r=2.5 \sigma$; between $r=2.206 \sigma$ and $r=2.5 \sigma$, the potential and its first derivative are continuously decreased to zero^28^. We use LJ units to describe our results. Table 1 gives these units for several exemplary materials. Note that the LJ potential is a poor description of tetrahedrally bonded materials like silica and water; here these values can only serve for an order-of-magnitude orientation.

The grains are amorphous with a radius of $R=28.8$ and have been constructed by rapidly quenching from the melt^29,30^ with a rate exceeding 0.007 in Lennard–Jones units^31^. We verified that the pair correlation function agrees with that published in the literature^32,33^. The grains are relaxed in an NVE ensemble with velocity-proportional damping for a LJ time of 460 in order to obtain relaxed surfaces. Then two grains are put into contact and again relaxed for a LJ time of 7000 with velocity-proportional damping and for a LJ time of 2300 without friction; this establishes an adhesive neck as shown in Fig. 7a. In the following we denote the axis connecting the centers of the two grains as the *central axis*; its midplane is the *contact plane*. In order to allow for flexibility in modeling the adhesion of the two grains, the interaction between atoms of different grains is changed from the value $\epsilon$ to a reduced value $\epsilon _{12}$. This allows us to check the dependence of several contact quantities—such as the adhesive force, “Adhesive force” section, and the rolling torque, “Rolling” section—on the specific surface energy. The value of $\epsilon _{12}$—denoted as the *intergrain attraction*—is varied between 0.25 and 0.75 in units of $\epsilon$. We choose $\epsilon _{12}$ to be below $\epsilon$ to ensure that the intragrain interaction is stronger than the intergrain interaction. This helps to avoid significant intermixing of grains and allows for more pronounced rolling and twisting motions.

For each value of $\epsilon _{12}$, three intergrain contacts are prepared by rotating the grains arbitrarily before contacting them, in order to determine the contact area and adhesive force. For the rolling and twisting simulations, only one of these contacts is used since the contact area changes dynamically throughout the simulation due to grain rotation.

**Figure 2:**
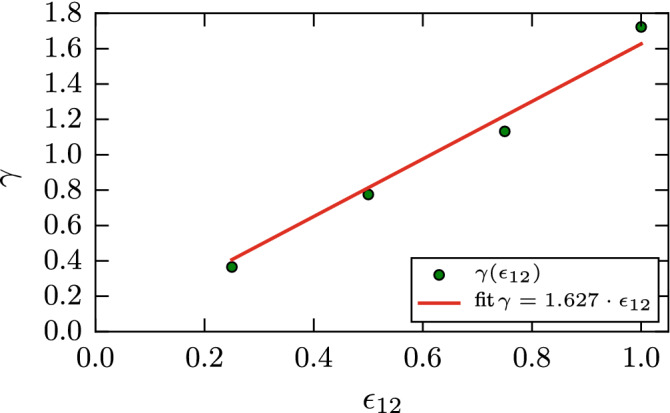
Dependence of the surface energy $\gamma$ on the intergrain attraction $\epsilon _{12}$.

Since the intergrain attraction is changed with respect to the intragrain attraction, we determine the specific surface energy, $\gamma$, of our grains. To this end, we prepare a cubic sample of amorphous Lennard–Jones material and measure the difference in energies between a system with periodic boundaries in all directions and a system in which a free surface has been introduced. We use an NPT integrator and relax the system to a temperature of $10^{-3}$ and pressure $<10^{-3}$. The surface energy can be calculated as the difference in potential energy of the two systems divided by twice the area of a side of the cube. Figure 2 shows that the surface energy is to a good approximation linear in the intergrain attraction $\epsilon _{12}$,

2 $$\begin{aligned} \gamma = 1.627 \epsilon _{12}. \end{aligned}$$

The molecular dynamics simulations are performed with the LAMMPS^34^ code. The elastic properties of our sample were determined using the ELASTIC module within LAMMPS. We obtain a Young’s modulus of $E=46.4$ and a Poisson ratio of $\nu =0.37$, from which we determine the indentation modulus $E_{\mathrm{ind}}= E/(1-\nu ^2)= 53.5$ and the shear modulus $G=16.9$; these results are in good agreement with previously published data on amorphous Lennard–Jones material^35^. Atomistic snapshots are rendered using the software OVITO^36^.

**Table 1:** LJ parameters, $\epsilon$ and $\sigma$, and mass, *m*, for four materials: Ar^37–39^, Ag^39–41^, silica^42^ and water^42^.

	$\epsilon$ (meV)	$\sigma$ (Å)	*m* (amu)	${\bar{v}}$ (m/s)	${\bar{t}}$ (ps)
Ar	10.32	3.41	39.95	158	2.16
Ag	345	2.644	107.87	555	0.476
SiO$_2$	221	2.39	60.09	596	0.401
H$_2$O	53	3.19	18.02	533	0.599

LJ units for velocity, ${\bar{v}}=\sqrt{\epsilon /m}$ and time ${\bar{t}}=\sigma \sqrt{m/\epsilon }$.

## Results and discussion

### Adhesive force

**Figure 3:**
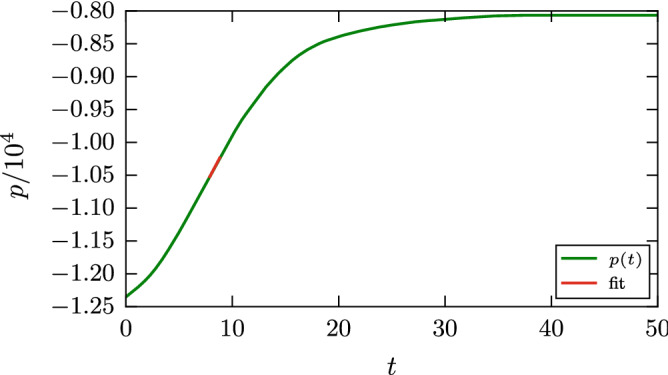
Temporal evolution of the relative momentum *p* in a separation simulation of two grains at an initial value of the relative velocity of $v=0.247$ with $\epsilon _{12}=0.75$.

A basic quantity characterizing the interaction of grains is the adhesive force, $f_{\mathrm{adh}}$. It is defined as the force needed to separate two grains. DMT theory gives

3 $$\begin{aligned} f_{\mathrm{adh}}= 2 \pi \gamma R, \end{aligned}$$

and JKR substitutes the prefactor 2 by 1.5 in this law^43^. There may be several ways to calculate $f_{\mathrm{adh}}$ in molecular dynamics; we chose an approach that is in the spirit of our determination of the damping torques. We give each grain a momentum along the central axis in opposite direction and monitor the temporal evolution of the momentum *p* of the two grains. Figure 3 gives an example of such a simulation. the two grains separate after around $t=40$. We determine $f_{\mathrm{adh}}$ as the maximum slope in this graph (highlighted as ‘fit’ in Fig. 3), since this gives the maximum attractive force that the two grains exert on each other.

**Figure 4:**
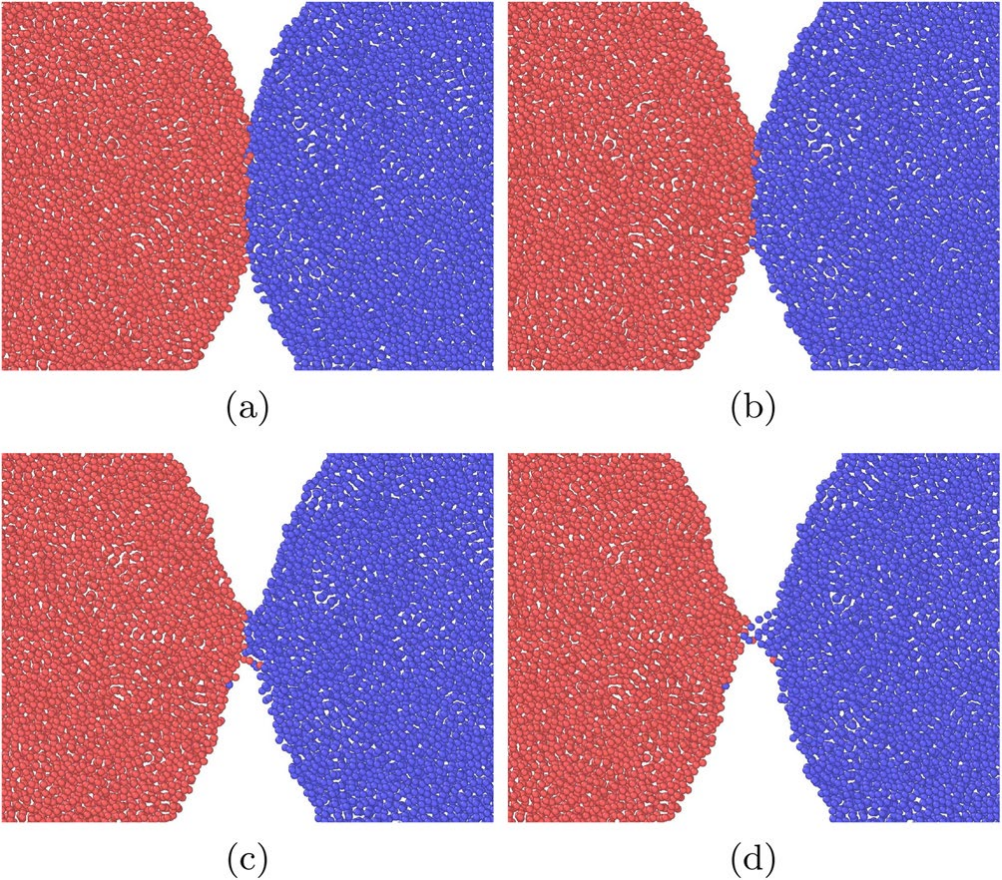
Snapshots of a separation simulation with $\epsilon _{12}=0.75$ for an initial value of the relative velocity $v=0.247$. Snapshots are spaced by $\Delta t=9.3$. In all subpanels, the grains are aligned such that the central axis lies horizontally in the middle of the figure.

Figure 4 shows snapshots for this case; they show cross-sectional views of a central slice of thickness 5.88 containing the contact axis. Upon separation, the contact surface deforms into a thin adhesive neck which is elongated until it tears. The force maximum occurs at around $t=10$, corresponding to Fig. 4b, indicating that it is the early stages of the work of neck formation that determine the adhesive force. Neck extension and narrowing cost less work.

**Figure 5:**
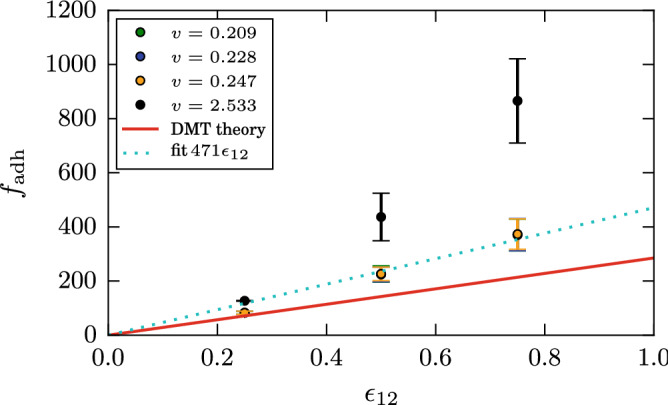
Dependence of the adhesive force $f_{\mathrm{adh}}$ on the intergrain attraction $\epsilon _{12}$. DMT follows the law Eq. (3). The dashed line gives a linear fit through the data for the low separation velocities *v*.

Figure 5 assembles the data of $f_{\mathrm{adh}}$ obtained for various values of the intergrain attraction $\epsilon _{12}$; for each $\epsilon _{12}$, 4 different separation velocities *v* in the range of $v=0.2$–2.5 have been simulated for three different intergrain contacts. For the low velocities, $v=0.2$–0.25, the values of $f_{\mathrm{adh}}$ nicely coincide. However, for the highest velocity simulated, $v=2.533$, the values of $f_{\mathrm{adh}}$ are considerably increased. An inspection of the snapshots taken for this event shows that in this case, no adhesive neck forms between the two grains; rather they separate quickly, and also the maximum slope of the *p*(*t*) curves occurs quite early in the separation process, when all bonds are more or less simultaneously broken.

The simulation data may be compared to the prediction of DMT theory, Eq. (3), in Fig. 5. For the lowest intergrain attraction, $\epsilon _{12}=0.25$, the agreement is satisfactory for the low separation velocities. However, with increasing $\epsilon _{12}$, the simulation shows that larger forces are necessary to overcome the attractive forces. These are necessary, since the processes extending and eventually breaking the adhesive neck occur in non-equilibrium, while DMT assumes that the breaking of the contact occurs in a quasi-static way, where the neck geometry can adapt instantaneously to the separation of the grains. The simulation data may be fitted by a linear law, $f_{\mathrm{adh}}= \alpha \epsilon _{12}$ with $\alpha = 471$, while DMT theory yields a prefactor of $\alpha = 285$, below our results. The adhesion forces for the largest separation velocity show a superlinear increase with intergrain attraction.

We conclude that our method of determining $f_{\mathrm{adh}}$ using molecular dynamics simulations provides results in satisfactory agreement with available macroscopic theories even though our grains are of nanoscopic size. For larger separation velocities and intergrain adhesion, deviations show up.

### Rolling

In order to initiate a purely rolling motion of the two grains, we start the simulation by giving both grains an initial angular velocity ${\omega }/2$, parallel to the contact plane, of the same absolute value but in opposite directions, see Fig. 1a. This is done by assigning each atom a velocity of $\mathbf {v}=\frac{1}{2} {\omega }\times \mathbf {r}$. The ensuing dynamics is then simulated in an NVE ensemble. We consider initial angular velocities of $\omega = 0.013$, 0.027, and 0.040.

**Figure 6:**
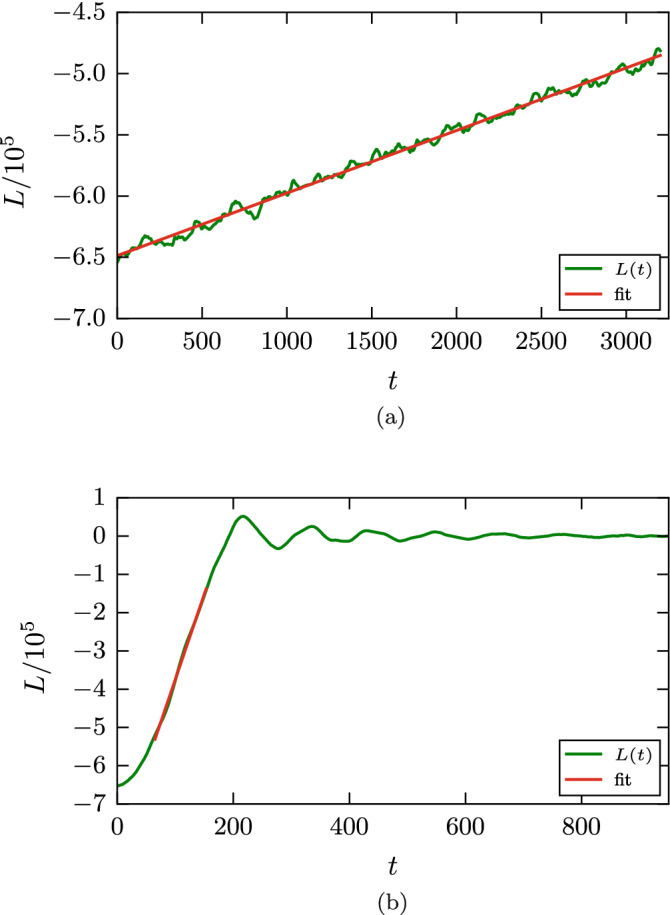
Rolling simulation: Temporal evolution of the angular momentum *L* for an intergrain attraction $\epsilon _{12}=0.25$ (**a**) and $\epsilon _{12}=0.75$ (**b**) for an initial value of the angular velocity $\omega =0.04$.

**Figure 7:**
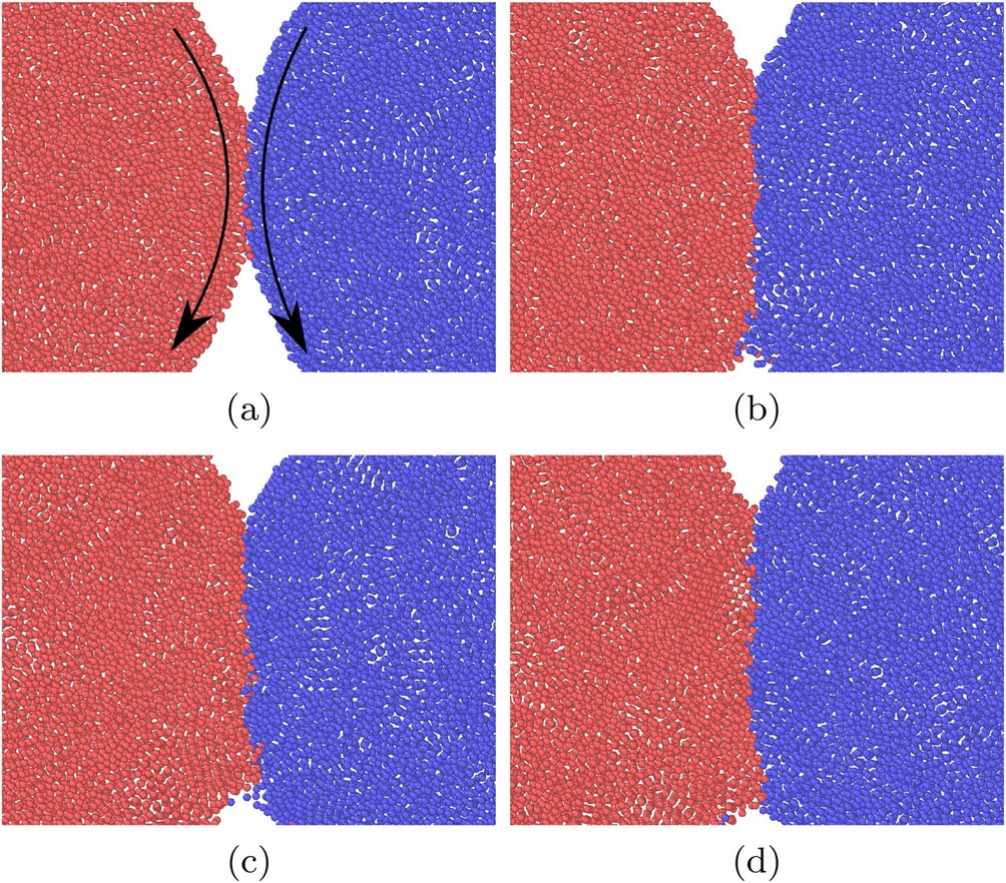
Snapshots of a rolling simulation with $\epsilon _{12}=0.75$ for an initial value of the angular velocity $\omega =0.04$. Snapshots (**a**, **b**, **c**, **d**) are spaced by $\Delta t=46.4$. The arrows in (**a**) indicate the rotation direction, see Fig. 1a. In all subpanels, the spheres are aligned such that the central axis lies horizontally in the middle of the figure.

We monitor the time evolution of the angular momentum of each grain, which is calculated via $\mathbf {L}=\sum \mathbf {r}\times \mathbf {p}$. Figure 6 gives two examples of the time evolution of the angular momentum. For a considerable part of the simulation we find a linear change in angular momenta. The damping torque $d_{\mathrm{roll}}$ is calculated from the slope of the *L*(*t*) curve. Here and in the following section, the slope is determined by fitting *L*(*t*) to a linear law in the range where *L* is between 20 % and 80 % of its initial value; only for the smallest value of $\epsilon _{12}$, where *L* does not decrease entirely to 0 but follows a linear law nicely, cf. Fig. 6a, the entire time range is used for the fit. Note that for the smallest angular velocity, Fig. 6a, the time evolution of the angular momentum is not monotonous; this is caused by the roughness of the surface of the two grains, and thus of the newly forming contact zone. Such fluctuations are absent for stronger intergrain attraction due the broad adhesive neck forming between the two grains. This simulation is visualized in Fig. 7. During the rolling motion, new surface enters the contact zone, which as a consequence both widens and roughens. In the case shown, the radius of the contact zone increases by a factor of around 2. We furthermore observe that the contact zone becomes asymmetric during the simulation shown in Fig. 7, such that it becomes extended in front of the rolling contact and shortened behind it. For smaller values of $\epsilon _{12}$, this asymmetry is less pronounced.

When the rolling process ends, the two grains still perform rolling oscillations, in which the angular momentum changes sign periodically, see Fig. 6b. This oscillation is quickly damped out.

**Figure 8:**
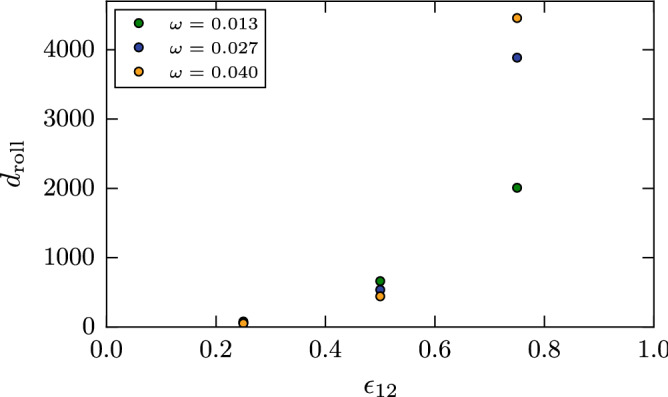
Dependence of the damping torque $d_{\mathrm{roll}}$ on the intergrain attraction $\epsilon _{12}$.

The damping torques calculated for all our simulations are assembled in Fig. 8. While the influence of the initial angular momentum on the torque is small for the two smaller values of the intergrain attraction, it is sizable for the highest value of $\epsilon _{12}=0.75$; here an increase of $d_{\mathrm{roll}}$ with $\omega$ shows up. Figure 7, which shows snapshots for the highest value of $\omega$, demonstrates that this $\omega$ dependence is caused by the dynamic changes in the contact zone induced for high rotation speeds.

Continuum models of the rolling torque^13,23,24^ attribute the damping torque to the dynamic form of the adhesive neck during rolling. As new material enters the contact zone at the front end and the contact area releases material at the rear end, an asymmetry between the front and the back part of the contact zone is established. It is described by the *asymmetry length* $\xi$ which quantifies the distance of the center of the contact zone to the central axis, i.e., the axis connecting the centers of the two grains. Using this quantity, the damping torque of rolling motion can be described as

4 $$\begin{aligned} d_{\mathrm{roll}}=2f_{\mathrm{adh}}\xi . \end{aligned}$$

This formula thus formulates the lever rule based on an adhesive force $f_{\mathrm{adh}}$ acting along the central axis and a lever arm $2\xi$ perpendicular to it.

**Figure 9:**
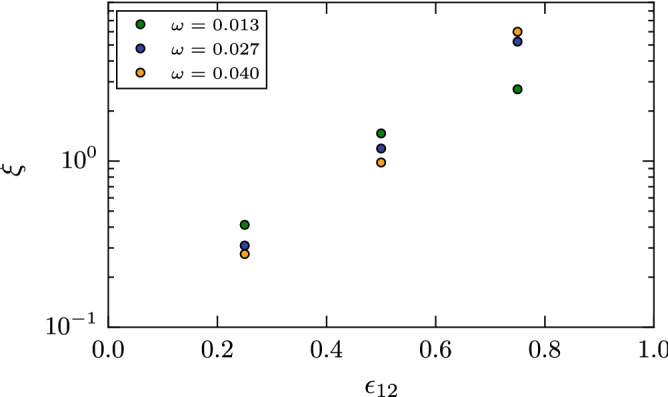
Dependence of the asymmetry length $\xi$ on the intergrain attraction $\epsilon _{12}$.

In our simulations, the contact zone fluctuates strongly during the rolling process (cf. Fig. 7) such that we could not determine $\xi$ directly from our simulations. We therefore use Eq. (4) to determine this asymmetry parameter from our simulation results for $d_{\mathrm{roll}}$ and $f_{\mathrm{adh}}$. The results are displayed in Fig. 9; here we used the adhesive force as determined for the smallest separation velocity such that the spread in the $\xi$ values obtained is due to the various rotational velocities used. The dependence of the results on angular velocity thus is inherited from $d_{\mathrm{roll}}$, Fig. 8. For the two smaller values of the intergrain attraction, $\xi$ decreases with $\omega$, i.e., for quick rotation, the contact zone has not sufficient time to develop a sizable asymmetry. For the highest value of $\epsilon _{12}$, though, the situation is reversed such that a swifter rotation induces a stronger asymmetry.

The asymmetry length $\xi$ shows an exponential dependence on the intergrain attraction, changing its value by almost an order of magnitude when $\epsilon _{12}$ changes by a factor of 3, from $\epsilon _{12}=0.25$ to 075. This increase is responsible for the superlinear behavior of the damping torque on $\epsilon _{12}$ in Fig. 8. This strong dependence of $\xi$ on $\epsilon _{12}$ is surprising in view of the fact that $\xi$ was originally introduced^13^ as a materials parameter of the order of one interatomic distance that is independent of the contact dynamics. Our results now demonstrate that this materials parameter sensitively depends on the surface characteristics of the grains, i.e., on the surface energy $\gamma$ (which we parameterize by $\epsilon _{12}$). From a physics point of view, this dependence is not surprising: for a large surface energy, and hence large intergrain attraction, the contact zone will be strongly affected by the dynamic contact during the rolling process, and hence the asymmetry length will increase.

Literature data on $\xi$ are available for silica spheres. Here the older literature^23^ recommended a small value of $\xi =1$ Å, while experimental measurements obtained a value that is more than one order of magnitude larger, $\xi =32$ Å^18^. Using Table 1, these values translate to $\xi = 0.3$ and 9.4 in LJ units; these values are indeed contained within the data assembled in Fig. 9. Our results thus show that the two literature values for silica grains may both be realistic, depending on the surface quality of the grains. It is known that due to adsorbate layers and passivation of dangling bonds of silica surfaces, the surface energy of silica grains strongly depends on the environment^20^. Note that values of $\xi$ below or around 1 are of the order of one nearest-neighbor distance in the material, while larger values denote an asymmetry length spanning a size of several atoms distance. This effect is caused by the substantial broadening of the contact zone observed in the case of the largest $\epsilon _{12}$, which also allows for a larger asymmetry to develop.

We conclude that the damping torque of rolling motion is strongly superlinear in the intergrain attraction. This superlinearity can be traced back to a strong dependence of the asymmetry length—the ‘lever arm’ in applying the damping torque—on $\epsilon _{12}$, which is caused by the dynamic contact of the rolling grains. For an exact prediction of $\xi$, a good knowledge of the surface energy of the grain is important.

### Twisting

**Figure 10:**
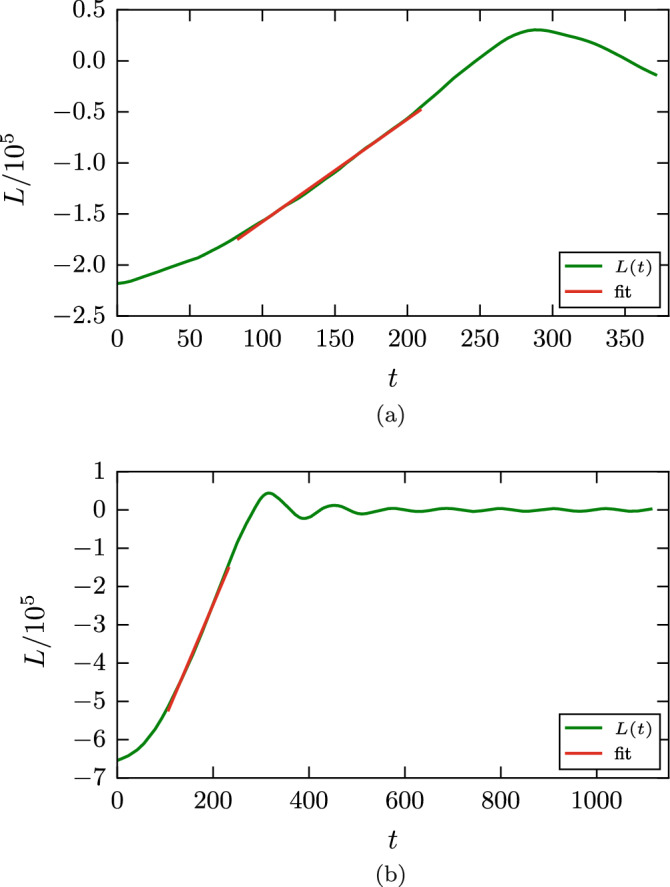
Twisting simulation: Temporal evolution of the angular momentum *L* for an initial angular velocity $\omega =0.013$ (**a**) and $\omega =0.04$ (**b**) for an intergrain attraction $\epsilon _{12}=0.75$.

The twisting simulations are performed analogously to the rolling simulations, but with an angular velocity oriented perpendicular to the contact plane, see Fig. 1b. The data analysis is analogous to that for the rolling case, such that we monitor the time dependence of the angular momentum during twisting motion, Fig. 10. Again we observe a roughly linear decrease of the angular momentum with time, such that we can extract a damping torque $d_{\mathrm{twist}}$. Note that, similar to the rolling case, after the twisting rotation is stopped, the two spheres experience a small number of twisting oscillations around the central axis, which are quickly damped out.

**Figure 11:**
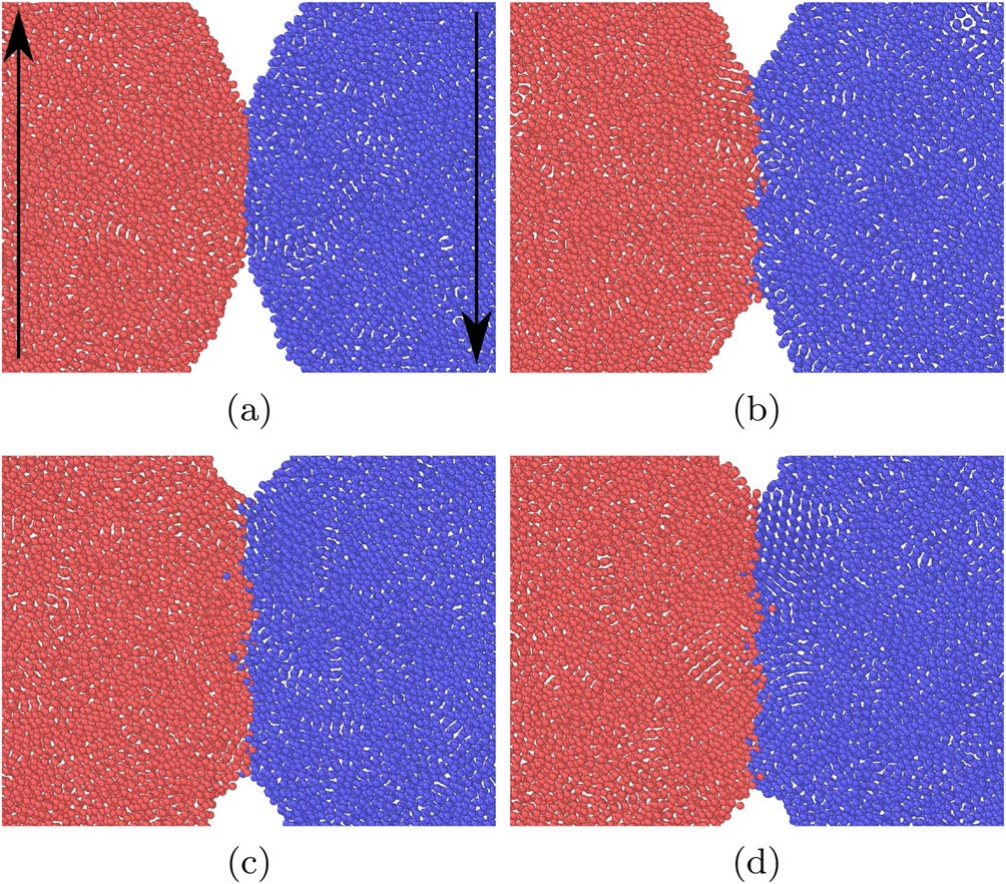
Snapshots of a twisting simulation with $\epsilon _{12}=0.75$ for an initial value of the angular velocity $\omega =0.04$. Snapshots (**a**, **b**, **c**, **d**) are spaced by $\Delta t=74.2$. The arrows in (**a**) indicate the rotation direction, see Fig. 1b. In all subpanels, the grains are aligned such that the central axis lies horizontally in the middle of the figure.

Figure 11 shows snapshots of the time evolution of the contact zone during twisting motion. A strong widening of the contact zone is seen and it becomes rough, as atoms from both grains are pushed into the other grain.

**Figure 12:**
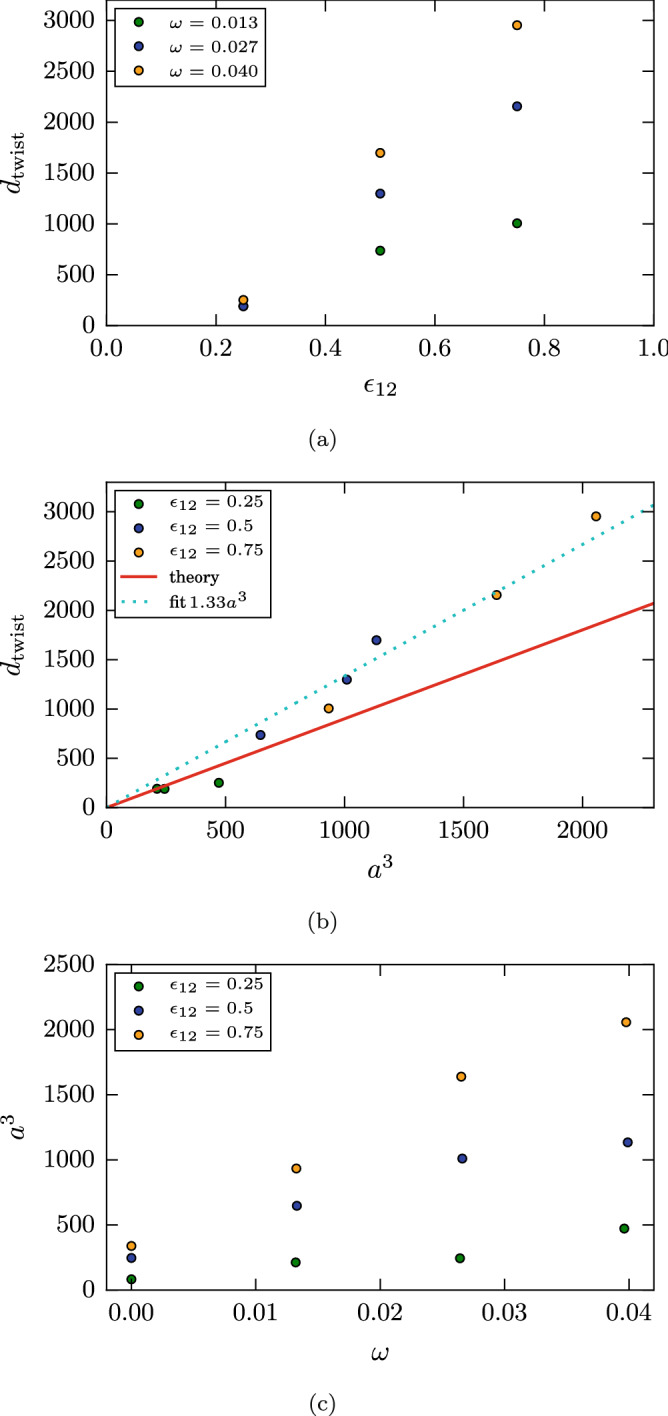
Dependence of the damping torque $d_{\mathrm{twist}}$ (**a**) on the intergrain attraction $\epsilon _{12}$, and (**b**) on the cube of the contact radius *a*. The full line gives the theory result, Eq. (5), and the dashed line gives a linear fit. (**c**) Dependence of the cubic contact radius on the angular velocity $\omega$.

Figure 12a gives a synopsis of the dependence of the twisting torque on intergrain adhesion and rotation speed. A strong increase of $d_{\mathrm{twist}}$ with both $\epsilon _{12}$ and $\omega$ is observed. Available theories^13,25^ of the twisting torque calculate the torque from the sliding friction of the two grains above each other. This friction arises from (atomic) surface roughness, such as from surface steps in the case of crystalline grains or from the inherent atomistic roughness of amorphous grains; an additional term comes from the sliding friction of two flat surfaces that is caused by atomistic stick-slip processes. Dominik and Tielens^13^ calculate the first contribution to the twisting torque as

5 $$\begin{aligned} d_{\mathrm{twist}}= \frac{G}{6\pi } a^3, \end{aligned}$$

where *G* is the shear modulus of the grain material and *a* the contact radius. This formula is often used in granular mechanics calculations^10,14–17^, neglecting the second, stick-slip contribution.

The contact radius *a* is determined in our simulations as follows. We calculate the number of atoms $N_c$ in a sheet of width *d* centered on the contact plane. Using the atom number density $n=1.00$ of our sample, we obtain *a* from $N_c= \pi a^2 dn$. As due to the small opening angles of our contact zones, see Figs. 7 and 11, $N_c$ depends on *d*, we calculate $N_c$ for several widths *d* and extrapolate to $d\rightarrow 0$. As the contact radius evolves in time during the twisting, we use its average value during the interval of time when the maximum torque is established.

The obtained data can be compared to the prediction of JKR theory^3,44^,

6 $$\begin{aligned} a_{\mathrm{JKR}}= \left( \frac{9\pi R^2 \gamma }{2 E_{\mathrm{ind}}} \right) ^{1/3}. \end{aligned}$$

Here the so-called indentation modulus $E_{\mathrm{ind}}= E/(1-\nu ^2)$ is determined from Young’s modulus *E* and the Poisson ratio $\nu$. As Table 2 demonstrates, our simulation result are in satisfactory agreement with the theoretical prediction, Eq. (6), and feature in particular the mild $\gamma ^{1/3}$ increase predicted by JKR. We note that the DMT result for the contact radius is smaller by a factor of $3^{1/3}=1.44$ than $a_{\mathrm{JKR}}$^3,44^.

**Table 2:** Contact radius *a*, as obtained from our simulations, compared to the JKR prediction, $a_{\mathrm{JKR}}$, Eq. (6).

$\epsilon _{12}$	0.25	0.50	0.75
*a*	$4.35 \pm 0.15$	$6.27 \pm 0.53$	$6.97 \pm 0.95$
$a_{\mathrm{JKR}}$	4.5	5.6	6.4

Simulation data are averages over three relaxed contacts.

Figure 12b plots the twisting torque versus $a^3$ to test Eq. (5). For the smallest intergrain adhesion, $\epsilon _{12}=0.25$, the torque is well described by theory, Eq. (5). For higher adhesion, the torque is above the prediction; however, the data still align on a line $d_{\mathrm{twist}}=\beta a^3$ with $\beta =1.33$ rather than the JKR value of $\beta =G/6\pi = 0.90$, Eq. (5).

The Dominik-Tielens^13^ theory of damping torques presupposes that the contact radius does not change during the twisting contact. This is not the case in our simulations where the contact radius *a* increases with angular velocity for all values of $\epsilon _{12}$, see Fig. 12c. The physical reason of this increase was discussed with Fig. 11. This effect needs to be included in Eq. (5) in order to get a consistent description of the twisting torque.

We conclude that the damping of twisting motion follows the theoretical prediction of a linear increase with the cube of the contact radius, if the instantaneous contact radius—which increases under twisting motion—is used. This effect of a dynamically increased contact radius will be important for soft grain materials, but appears hard to predict unless by simulations.

## Conclusions

We used molecular dynamics simulation of amorphous LJ grains to study the applicability of macroscopic contact theory to describe rolling and twisting motion of nm-sized nanoparticles. The adhesive force is in good approximation proportional to the surface energy, and follows roughly the DMT theory of adhesive contact. The asymmetry length $\xi$ responsible for the damping torque in rolling motion—characterizing the asymmetry of the adhesive neck during rolling motion and serving as the ‘lever arm’ of the torque—strongly increases with the surface energy of the grains. As a consequence, the damping rolling torque is strongly superlinear in the surface energy. Since this quantity may be strongly affected by surface chemistry (adsorbate layers) and is not always well known, this dependence adds to the uncertainty of modeling the rolling torque.

Also twisting motion is satisfactorily described by the law of Dominik and Tielens^13,25^ based on sliding friction. Since the damping torque of twisting motion increases with the cube of the contact radius *a*, a careful determination of *a* is important to verify this dependence. Our work shows that *a* increases dynamically under the torsional shear during the twisting rotation.

We conclude that our atomistic study basically validates important features of the available theories of rolling and twisting contacts—such as the linearity of the adhesive force with the surface energy and the $a^3$ dependence of the twisting torque. It also shows that the asymmetry length $\xi$ that is needed to quantify the rolling torque depends sensitively on the grain material, in particular its surface energy. Since besides comparison to experimental data^18,45^, atomistic simulation seems to be the only way to determine this quantity, further simulation studies of this parameter—in particular for realistic grain materials such as silica and water ice—will be welcome.

The good agreement between the atomistic results and macroscopic theories may appear astonishing in view of the fact that the grain radii amount to only around ten nm ($R \sim 30$ in Lennard–Jones units). However, already Luan and Robbins^2,26^ used small LJ spheres to study contact properties (adhesion and friction) and found generally good agreement between their atomistic results and continuum theories. They also pointed out that atomistic surface roughness, such as the one existing in the amorphous spheres used in the present study, seems to be the major reason for deviations from continuum results. Since the length scale of atomistic surface roughness is of the order of the interatomic distance, deviations from the continuum results will increase with decreasing sphere radius.

In the past, atomistic simulation was used to study energy dissipation in the normal motion of two grains, and also in sliding motion, and to compare to macroscopic continuum models^7,39^. Such studies profited also from their relevance to tribological issues, where contact between surface asperities rather than grains is of interest^2–4,44^. In contrast, rolling and twisting motion of two grains do not have an immediate analogy in tribological systems—if macroscopic tribosystems such as ball bearings are disregarded. Hence our study is of most relevance for the field of granular mechanics, and in particular to the modeling of intergrain contacts^9^. Nanoscopic grains are of particular interest in space applications^46,47^, such as in the modeling of protoplanetary dust discs^15,16,48^.
